# Feature Representation and Data Augmentation for Human Activity Classification Based on Wearable IMU Sensor Data Using a Deep LSTM Neural Network

**DOI:** 10.3390/s18092892

**Published:** 2018-08-31

**Authors:** Odongo Steven Eyobu, Dong Seog Han

**Affiliations:** 1 School of Electronics Engineering, Kyungpook National University, 80 Daehak-ro, Buk-gu, Daegu 41566, Korea; sodongo@knu.ac.kr; 2 School of Computing & Informatics Technology, Makerere University, Plot 56, Pool Road, P.O. Box 7062, Kampala, Uganda

**Keywords:** human activity recognition, data augmentation, feature representation, deep learning, long short term memory, inertial measurement unit sensor

## Abstract

Wearable inertial measurement unit (IMU) sensors are powerful enablers for acquisition of motion data. Specifically, in human activity recognition (HAR), IMU sensor data collected from human motion are categorically combined to formulate datasets that can be used for learning human activities. However, successful learning of human activities from motion data involves the design and use of proper feature representations of IMU sensor data and suitable classifiers. Furthermore, the scarcity of labelled data is an impeding factor in the process of understanding the performance capabilities of data-driven learning models. To tackle these challenges, two primary contributions are in this article: first; by using raw IMU sensor data, a spectrogram-based feature extraction approach is proposed. Second, an ensemble of data augmentations in feature space is proposed to take care of the data scarcity problem. Performance tests were conducted on a deep long term short term memory (LSTM) neural network architecture to explore the influence of feature representations and the augmentations on activity recognition accuracy. The proposed feature extraction approach combined with the data augmentation ensemble produces state-of-the-art accuracy results in HAR. A performance evaluation of each augmentation approach is performed to show the influence on classification accuracy. Finally, in addition to using our own dataset, the proposed data augmentation technique is evaluated against the University of California, Irvine (UCI) public online HAR dataset and yields state-of-the-art accuracy results at various learning rates.

## 1. Introduction

Current technological advancements in microelectronics have ushered in the design and manufacture of wireless miniature devices with key capabilities including the ability to house wearable inertial measurement unit (IMU) sensors, and wireless transmission capability. At the centre of this technological development is the desire for such miniature devices to be used in applications such as ambient assisted living (AAL) [1], physiological medical diagnostics [2], localization and navigation [3,4,5], mobile and wireless context-driven decision support systems [6], and security monitoring. The data that IMU sensors are capable of generating ranges from gyroscope, accelerometer, and magnetometer to global positioning system (GPS) information, depending on the product limitations. It is from this possibility of acquiring these IMU data that data-driven and knowledge-based learning models are relevant for data discrimination.

Deep learning models are data-driven learning models. Studies [7,8] have shown that deep learning models are able to learn and discriminate among human activities ranging from sitting, walking, climbing upstairs, walking down-stairs and falling, among others. However, studies [8] report that certain activities which produce relatively stationary data are challenging to discriminate amongst. Examples of such activities include sitting, standing and lying down (facing left, facing right, facing up and facing down) [9]. The challenge in discriminating relatively stationary data of different activities arises because of similarity of their feature representations. For example; when such data are considered as signals, transformations to the frequency domain may show relatively similar spectral information. It is for such a reason that proper feature extraction mechanisms are very important for classification purposes.

Statistical parameters and convolutions are the popular conventions for feature representation of data for learning purposes. Statistical parameters are popular for time-series data whereas convolutions are popular in image processing studies. However, various studies including [7,10] preferred to utilise a combination of statistical features and frequency domain features for data representation. Preferred combinations are always a choice geared towards improving the quality of the feature vector. To this end, the key point in a feature representation approach is in its quality that should represent the intrinsic characteristic of each class of data. Conventional approaches used for dimension reduction of data include principal component analysis (PCA) [11,12], and convolution approaches using defined kernels [7].

In this paper, a feature extraction approach is proposed. The features are extracted from a spectrogram of the 3-dimensional (3D) raw acceleration and gyroscope data collected in a defined period of seconds. A set of least and largest values from the spectrogram is selected to represent the data abstraction referred to as the feature vector used to training the deep learning model. The least and greatest value set selection approach is a kind of dimension reduction approach applied to the feature vector without compromising much on the originality of the data in this paper.

A feature representation for a given class can only be considered exhaustive qualitywise if all or a huge percentage of raw data variants of the same class are represented. Apart from just having few samples of training data as a classification problem, it is also reported both in practice and literature [13] that sensors exhibit a displacement problem during the process of data collection and testing even when the sensor is put at the same human body position during data collection for training and testing. The displacement problem is one of the causes of false classifications. The obvious solution to this problem is collecting a lot of training data while accounting for sensor displacement. This is a very tedious process, hence a challenge in human activity recognition (HAR) studies. One powerful solution for this problem is to perform data augmentation.

Data augmentation provides an opportunity to create deformations of training samples without changing the semantics of the raw data. Such deformations are added to the training dataset with the objective of representing unseen raw data. Data deformation is a popular practice in image processing where transformations such as image rotation and image scaling pose similar significant semantic meaning of the original image. However; for wearable sensor data, it is very challenging to generate suiting deformations that maintain the semantics of the data label. This rigidity is because the data variations are an intensity factor of motion. Therefore, depending on the kinds of activities under study, a deformation may alter the semantic meaning of the label. Nevertheless, data augmentation has been successfully applied in wearable sensor data in studies such as [13,14] showing positive state-of-the-art results, and sound recognition [15]. It should be noted that data augmentation for wearable sensor data has not been exhaustively and systematically investigated unlike augmentations for image, sound and speech recognition. In this article, a data augmentation procedure for wireless IMU sensor data is proposed. The major data augmentation machinery used is the down-sampling approach based on local averaging. In addition to local averaging, data shuffling is done to cause data variations in the feature vector sets to enable further local averaging of the same data and to reduce on overfitting.

Apart from the feature representation as a challenge in classification problems, the number of classes to be dealt with in an experiment is also a challenge as classification results tend to be skewed to some class or classes due to data imbalance [16,17,18]. This phenomenon is common in binary classification [19,20] where only two classes are involved. However, the same challenge presents itself in the classification of three classes as well. In this paper, experiments are conducted to recognize only three activities with the objective of determining the influence of the proposed augmentation approach on the data imbalance and overfitting problem.

In this paper, the problem of classifying human activities using data from wearable IMU sensors is handled using a deep recurrent neural network (RNN) with long short term memory (LSTM). The LSTM neural network is tweaked with a greedy-wise hyper-parameter adjustment for the learning rates and feature vector size in order to understand the network performance.

The original contributions of this paper can be summarized as follows: (1) a proposed feature extraction algorithm whose abstraction is based on defined least and largest spectral values, (2) an ensemble of feature space augmentation methods applicable to wearable IMU sensor data and suitable for human activity classification and (3) an experimental analysis of both the proposed feature extraction and an ensemble of augmentations to understand the influence of each method on classification accuracy. The rest of this article is organized as follows: Section 2 discusses the related works in HAR and data augmentation. Section 3 presents the proposed feature extraction algorithm and augmentation method. Section 4 presents the experimental setup. Section 5 presents the results and discussions. Finally, Section 6 concludes this article.

## 2. Related Works

HAR using wearable IMU sensors exhibits possible diverse application areas. For this reason, HAR studies are very attractive nowadays. Already, various domain specific studies seeking to utilize HAR based on wearable sensors have been conducted and more are still emerging. Therefore, in this section, selected HAR studies are categorically reviewed with the interest of showing existing feature set selection and classification methods for time series data in the following order: (1) HAR for AAL (elderly care, human behaviour understanding), and (2) HAR for healthy living (fitness for preventive health care). Given the method applied in this article, a subsection with keen interest on feature representation and data augmentation studies in HAR that use deep learning is presented to end the current state-of-the-art and acts as an introduction to the proposed method in this article.

### 2.1. HAR for AAL

The vision of AAL is that persons should be able to live life independently regardless of their physical weakness and human disabilities. Assistive technologies are the key drivers behind this vision. Fall detection in elderly people [21,22,23] is seen to be the most popular and attractive application in AAL studies. Tremor caused by diseases like Parkinson’s have been quantitatively assessed in studies like [24] to aid quick diagnosis and remedies. The possibility of understanding human intention using motion data has also been studied in [25] as a build-up to achieve AAL.

In [21], both the Kinect sensor and a wearable motion-sensing device are used to detect falls. The combination is aimed at minimizing the number of false alarms. In their system, a small number of false alarms are achieved owing to visual validation of the fall alert generated based on motion data only. A total of 612 images were used for training from the University of Rzeszow (UR) fall detection dataset (URFD). For classification of the fall, the support vector machine (SVM) was used. The feature sets used for classification purposes and analysis included: the colour depth maps only, and colour depth + acceleration features. However, it should be noted that since the Kinect sensor is a vision based system, it is naturally limited by privacy controls. Cameras cannot be placed in some locations such as bathrooms, so when an elderly person falls while taking a bath in the absence of a camera, only the wearable sensor can be useful.

Mao et al. [22] proposed a fall detection method based on the acceleration and Euler angle data extracted from a wearable micro electro-mechanical system (MEMS) sensor to represent the orientation of the users’ body using Kalman solutions. Through experiments where the sensors were placed on the subjects’ shoulder, waist and foot, a threshold of acceleration was identified for accurate fall detection. In other words, the threshold of acceleration acted as the key feature for classification. However, despite of the accuracies achieved in the threshold approach, using adaptive thresholding would be more robust for fall detection. This is solely because different human beings have different structures naturally especially as they grow old. The natural body orientation of an elderly person may be somehow curved. Such cases would raise false alarms. Pierleoni et al. [23] proposed a fall detection algorithm which was implemented in a wearable device. Their system utilises a fusion of triaxial accelerometer, gyroscope and magnetometer data from an IMU sensor. Based on the root mean square (RMS), yaw, pitch and roll, the orientation of the subject can be specified to determine whether it’s a fall or not. In [24], a quantitative assessment of Parkinson’s tremor is conducted using least-square-estimation models. Tremor quantification was based on a time-frequency signal features got from IMU data.

Apart from using only inertial sensors for motion analysis, recent studies [26] have considered the fusion of both inertial sensors and camera vision sensors to improve on the human activity recognition accuracy. Vision-based activity recognition would require that feature extraction approaches for image data must be carefully considered to enable accurate activity detection. Various feature extraction approaches in image processing include using the spatio-temporal interest point (STIP) [27] detector, motion–energy images (MEI) and motion history images (MHI) [28].

Human gait modeling studies [29] have also come up with descriptive motion models which can be used to aid the recognition of persons and activities. Some descriptive gait models are based on imagery, animation and inertial sensor data to determine the human posture, motion, stride length, and stance time [30].

### 2.2. HAR for Healthy Living

Apart from selectivity in what to eat, a healthy body needs exercise to remain fit. Fitness-oriented applications focus on this need by being able to automatically identify human activities, log them and do statistical analyses for body fitness assessments [31]. In order to achieve the latter, wearable devices such as smart watches or smartphones are equipped with inertial sensors to generate data and user analysis software applications for feedback purposes [32]. Sports–related activities include swimming, jogging, walking, jumping, push-ups, running, playing football, roller skating among others. In [7,8], activities such as walking, standing, sitting, laying, walking upstairs, cycling, jogging were recognized based on a dep convolutional neural network (CNN) learning methodology. Basing on convolution theory, the features used in both studies are a result of applying defined filters to the original data set in [8] and to the spectrogram in [7].

In [33], a data analysis tool called *SwimMaster* was developed with the capability of identifying the swimming style, swimming stroke counter, body balance and rotation. Inertial sensors were mounted on the swimmers’ upper arm, the lower back and the right wrist for data collection purposes. Evaluation of the study parameters was based on an analysis of the yaw, roll and pitch values as discriminating features.

In summary, time series classification approaches as observed in the review above can be split in two major categories: (1) distance-based (SVM, *k* nearest neighbors (*k*-NN), least square estimations (LSE), Euclidean distances) and (2) feature-based methods (Fourier coefficients, spectrograms, logic regression, means, variance) being the fastest in training and most popular nowadays. The proposed classification approach in this article is feature-based with spectral information being extracted for classification.

### 2.3. Feature Representation Studies

Features are generally abstractions of data. The main purpose of feature extraction is to find abstractions from a data segment that can accurately represents the original data. In other words, the transformation of large input data into a reduced representation set of features, which can also be referred as feature vector, is called feature extraction [34]. Feature vectors include discriminating information between various activities or classes of data. It is the feature vectors that are used as inputs to classification algorithms. The Table 1 shows various feature representation approaches from various studies and their applications.

In Ravi et al. [7] the features are derived by first generating a spectrogram of the raw data and then performing a convolution process to generate the data abstraction which represents the feature vector. In contrast to Ravi et al. [7], the proposed feature representation approach in this article also uses spectrogram data and generates the data abstraction set of data based on defined least and greatest values of the same spectral data.

### 2.4. Data Augmentation Studies

Currently, there are indeed limited studies reported in literature that use or even address data augmentation as a mechanism for improving time series data classification accuracy for wearable sensors. Some of the existing methods are mentioned in this subsection. Guennec et al. [45] proposed window slicing and dynamic time warping (DTW). Cui et al. [46] as well proposed window slicing for data augmentation which is developed as follows: For a time series $T=\{t_{1},\ldots ,t_{n}\}$, a slice is a snippet of the original time series, defined a $S_{i:j}=\{t_{i},t_{i+1},\ldots ,t_{j}\}$, 1≤i≤j≤n. Suppose a time series T is of length n, and the length of the slice is s, the slicing operation will generate a set of n−s+1 sliced time series:

(1) $$Slicing(T,s)=\{S_{1:s,},S_{2:s+1},\;\ldots \;,\;S_{n-s+1:n}\}$$

where all the time series in Slicing(T,s) have the same label as their original time series T does.

In [14], a sizeable number of data augmentation methods for time series data are mentioned and implemented. These include:

(1) Rotations: To cater for multiple sensor placement scenarios which represent the same label, controlled data rotation may offer generalization ability of such unseen data. An example of such a scenario is when a sensor is placed upside down compared to its normal position during collection of training data.
(2) Permutation: This is a method to perturb the temporal location of with-in window events. To perturb the location of data in a single window, the data are first sliced into N same length segments. The segments are then randomly permutated to create a new window.
(3) Time-warping: Is another approach used to perturb the temporal location of data. This is done by smoothly distorting the time intervals between samples. This is like time scale modification (TSM) whereby the window can be compressed by reducing the time interval of samples or extended by increasing on the time interval between samples.
(4) Scaling: This approach involves changing the magnitude of the data in a window by applying a random scalar.
(5) Magnitude-warping: Involves changing the magnitude of each sample by convolving the data window with a smooth curve varying around one.
(6) Jittering: Involves including additive sensor noise.

It can be seen that most popular augmentations in literature are done in data space. However, recently, feature space augmentation approaches are being proposed. Some feature space augmentation studies are in studies like [47,48,49]. The synthetic minority over-sampling technique (SMOTE) by [49] is the most popular of these. In this article, we propose augmentation by local averaging which is further improved by data shuffling. Our augmentation approach is done in feature space and logically explained in the next section together with the feature extraction approach describing our proposed method.

## 3. Proposed Approach

**Figure 1:**
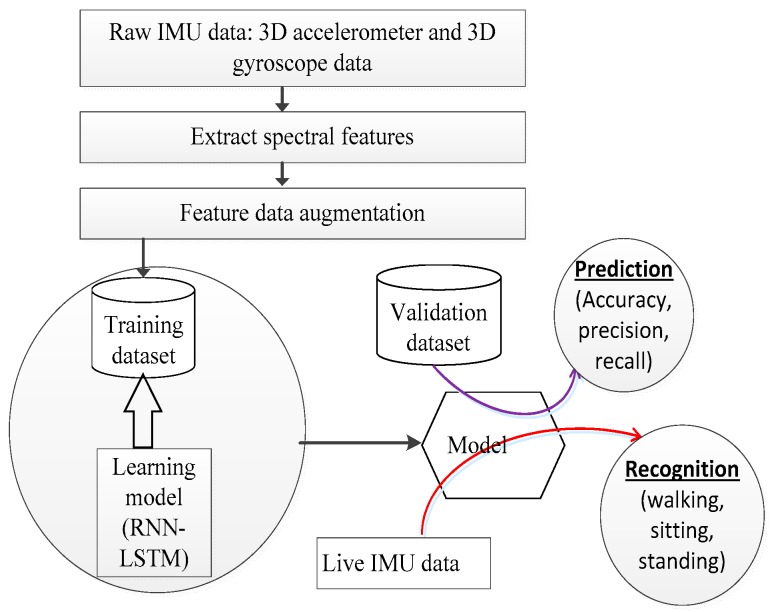
Human activity recognition system workflow.

This study is based on the paradigm illustrated by Figure 1. The contributions in this study are in the feature extraction block and data augmentation block. In the feature extraction block, the short Fourier transform (STFT) is used as a tool to generate a spectrogram from which the spectral information is extracted. The STFT is performed on IMU sensor raw data generated in a time window period of 3 s and later on its subsequent time overlaps. The spectral information is shaped as a vector which is reduced in length by sorting and extracting a set of defined least and greatest values hence forming the data abstraction. The manipulation to reduce on the size of the spectral information is motivated by the fact that large input feature vectors cause a long training time for a deep learning system compared to shorter feature vectors. The manipulated spectral feature vectors are finally used for training a deep learning system. The detailed description of the feature extraction process is explained in Section 3.1.

In the data augmentation block, two techniques are used. These are the local averaging as a down-sampling technique and shuffling. Local averages of the spectral feature dataset based on a defined criteria is calculated and then appended at the tail end of the feature set. Shuffling of feature vectors is done to create variation in the data in the case where further downsampling is desired. The detailed description of the augmentation procedure is explained in Section 3.2.

### 3.1. Feature Extraction Method

**Figure 2:**
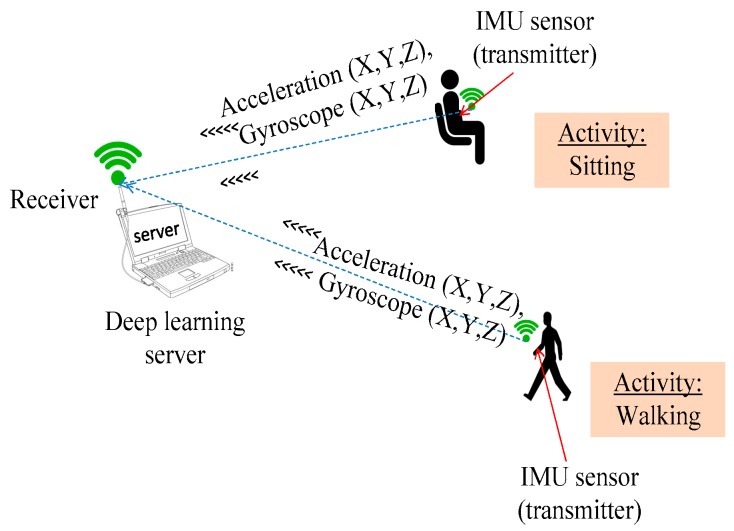
Data collection architecture.

**Figure 3:**
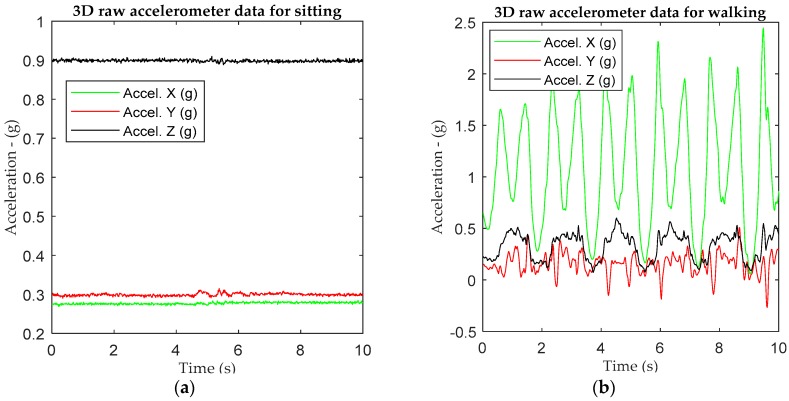
An example of 3D raw data for (**a**) sitting and (**b**) walking based on 1IMU sensor tied on the left-hand wrist.

**Figure 4:**
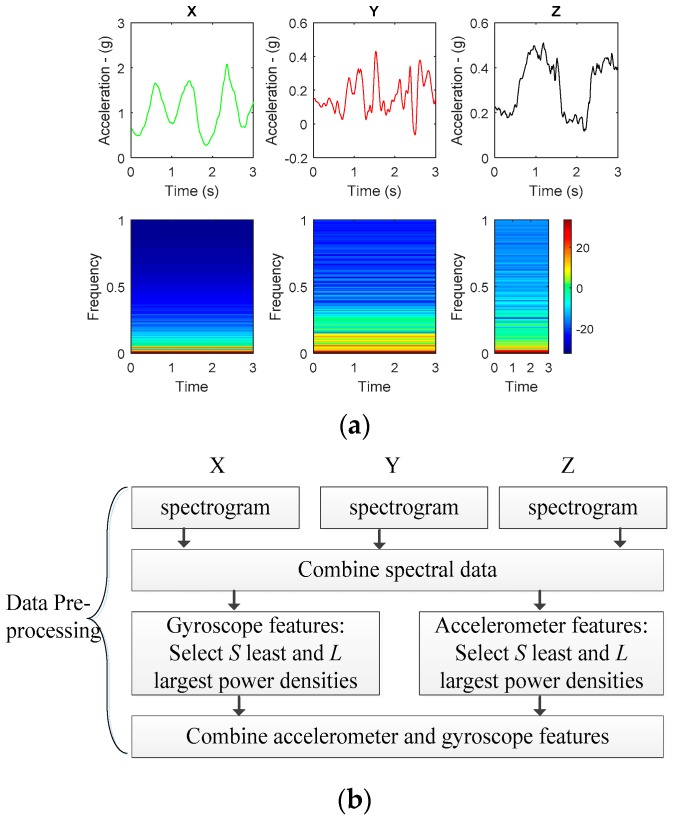
(**a**) An example of time domain data and their spectrogram representing walking data; (**b**) Proposed feature extraction algorithm.

Consider 3D accelerometer and gyroscope data extracted from an IMU sensor based on the experimental architecture in Figure 2. All triaxial accelerometer and gyroscope information from the IMU sensor are received at a server for learning and classification purposes. Figure 3a,b show the raw triaxial data extracts from sitting and walking scenarios in a period of 10 s. Figure 4a is an extraction of a traw riaxial walking signal from Figure 3 and showing its spectogram for a period of 3 s hence representing the interval for the activity recognition.

Figure 4b shows the workflow for the proposed feature extraction whose input are the spectrogram information generated from Figure 4a. It should be noted that subsequent windows from each of the Figure 4a data are generated by overlapping the previous window by 50% in order to generate other spectrograms for continuous feature extraction. Therefore, the STFT for each window extract can be generated by:

(2) $$\mathrm{STFT}\left\{k\left[n\right]\right\}\left(m,\omega \right)=\sum_{n=-\infty }^{\infty }k\left[n\right]W\left[n-m\right]e^{-j\omega n}$$

The spectrogram is then described as $|STFT\left(n,\omega \right){|}^{2}$ and k[n] can be any one of the 3D (*X* or *Y* or *Z*) raw signals at time n, m is the time shift applied to the window used in the transformation expressed as W[n]. For this study, the Hanning window was used and the number of data points used in each FFT block is 512 with a sampling frequency of 50 Hz. The Hanning window function is popularly used because a signal reconstruction close to the original signal is achievable.

At each window, when the spectrogram for each dimension is generated, the power densities in each spectrogram representation are all combined and sorted in an ascending order. A set of power densities comprising of the least and another set comprising of the largest power densities are considered as features of the data set. In this paper, the number of the least power denstities are referred to as S and the number of largest power densities are referred to as L. The generated S and L data for both the gyroscope and the accelerometer are combined ready for input to the learning module. Therefore, for this study, when S and L are each equal to 25 for both the accelerometer and gyroscope spectral data, then we generate a 100-feature vector dataset. In the same vain, when S and L are each equal to 50 for both the accelerometer and gyroscope spectral data, then we generate a 200-feature vector dataset. The 100-feature vector and 200-feature vector datasets are in this article referred to either as the initial feature set or our dataset in Section 5.

We are motivated to use the least and largest of the spectral information on the basis that spectrogram information in its raw form can be used to generate features using approaches like convolution successfully as seen in various literature such as [7]. In essence, by convolution, it is possible to extract the salient features in the spectrogram to represent the data. It is in the same vain that we can use the same raw spectral data without transforming it but carefully set the boundaries or limiting factors for selecting those to be used as features. The least and largest values of a specified size were used. Our approach to use the least and largest spectral value set for spectral data abstraction is motivated by the fact that the convolution process presents a larger latency compared to the selection process of least and largest spectral information. The latency factor is important when considering efficient real time processing.

### 3.2. Augmentation in Feature Space

**Figure 5:**
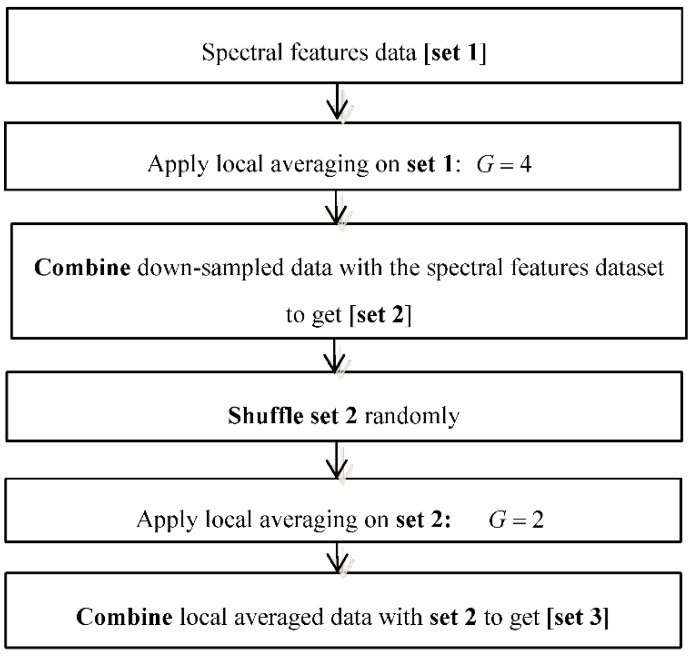
Data augmentation workflow.

**Figure 6:**
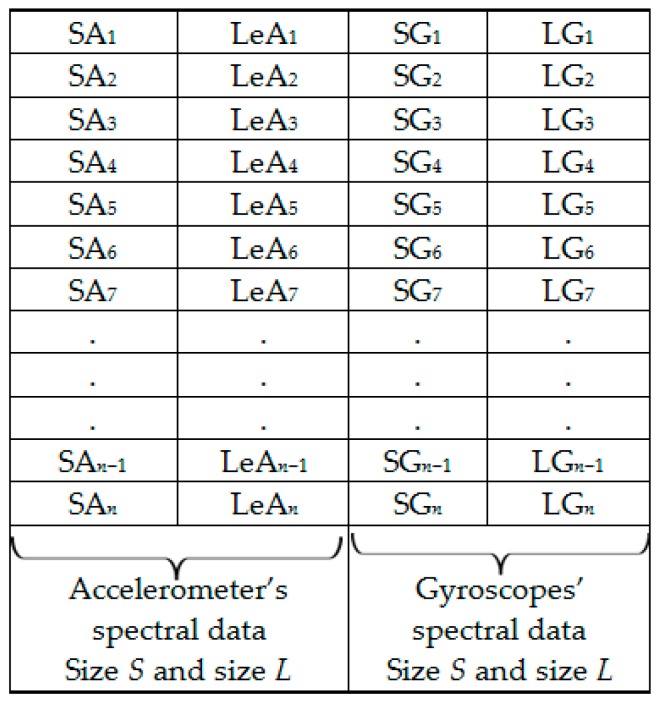
Set 1: OR dataset.

**Figure 7:**
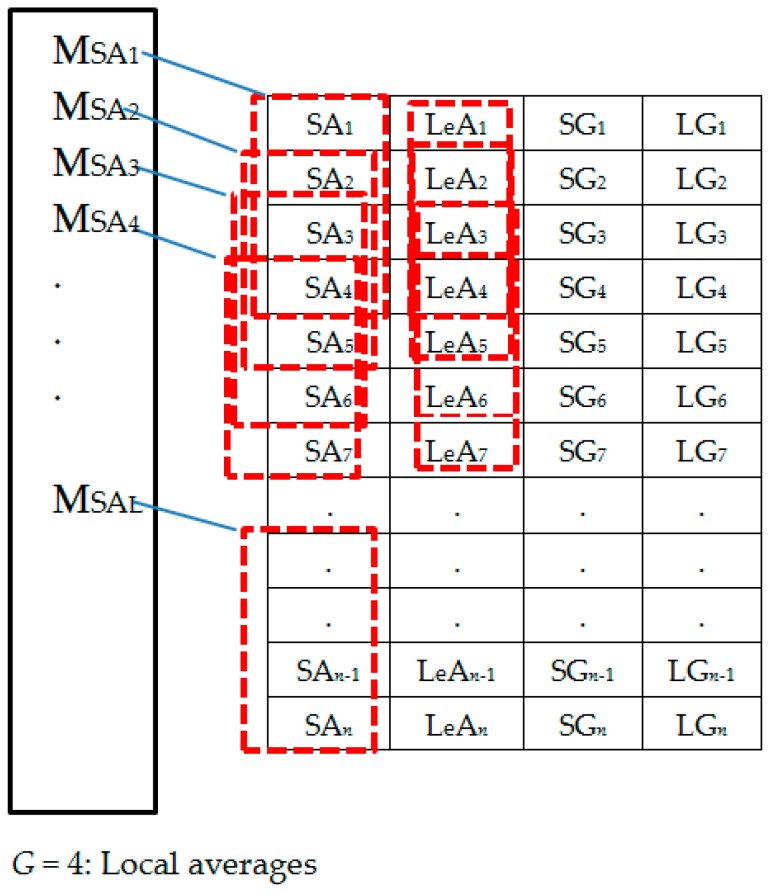
Set 1: Generating local averages.

**Figure 8:**
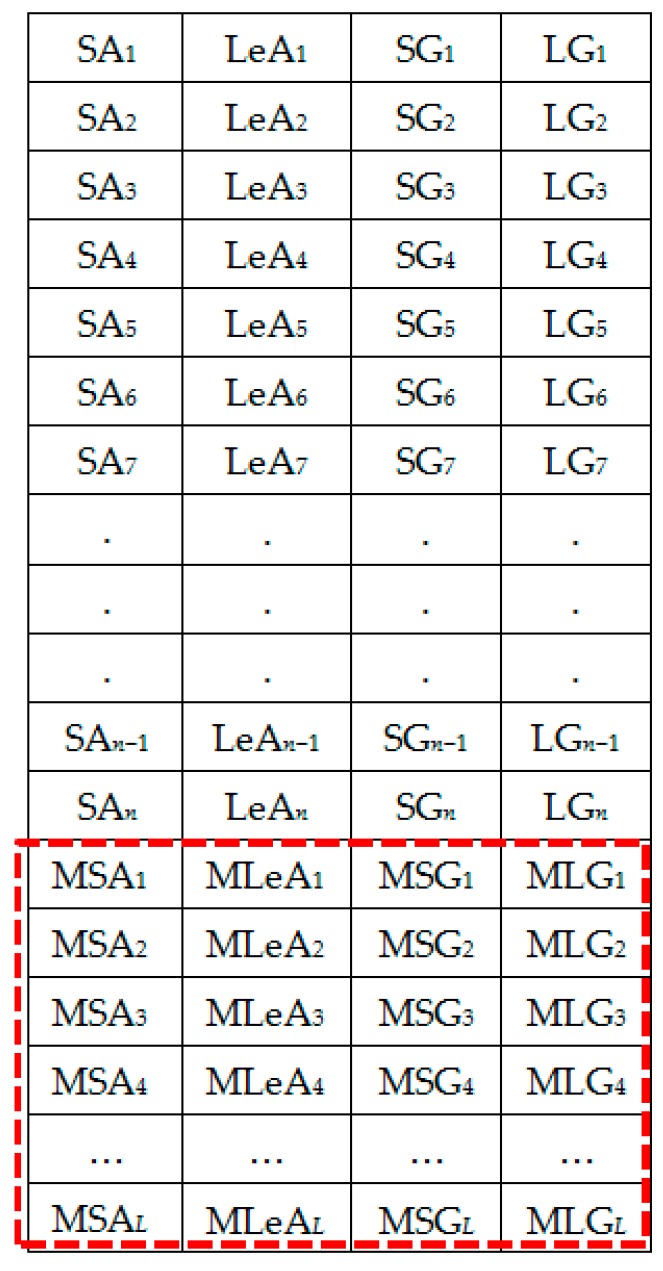
Set 2: OR + LA1 dataset.

**Figure 9:**
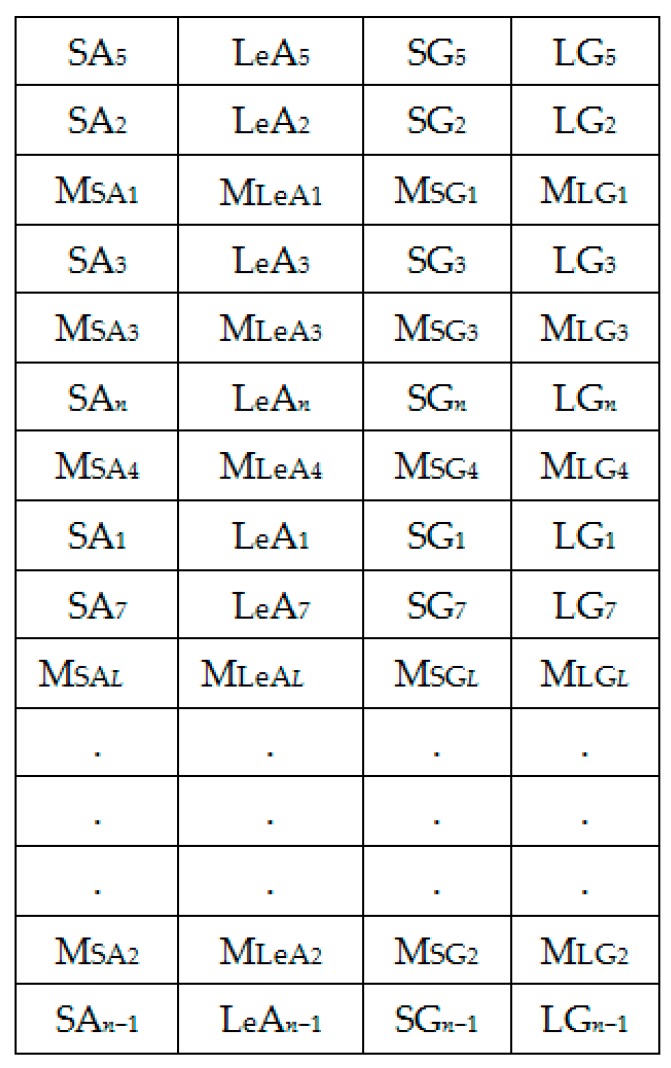
Randomly shuffled (OR + LA1 + SH) feature set 2.

**Figure 10:**
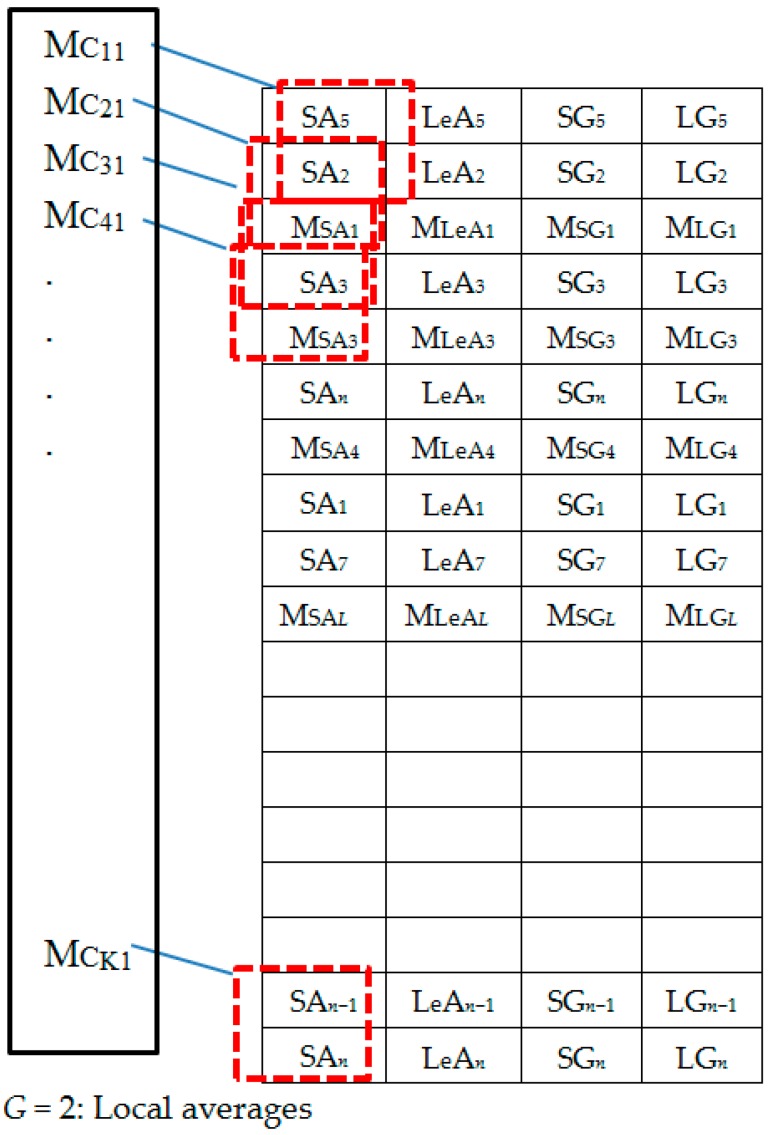
Generating local averages of the shuffled feature set.

**Figure 11:**
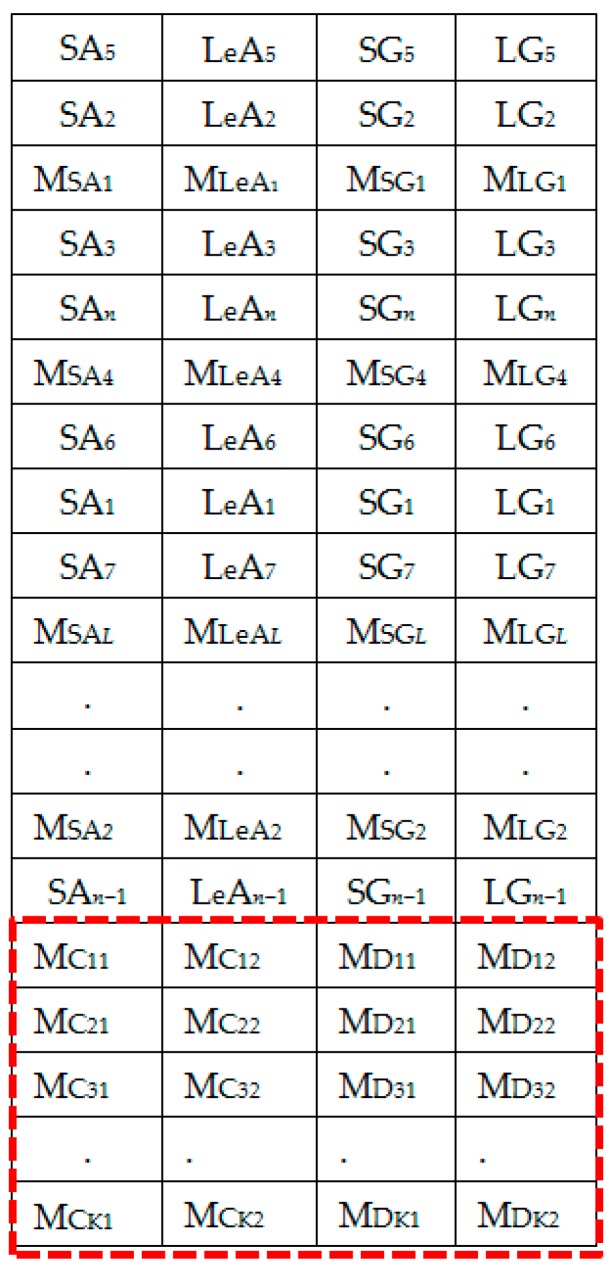
Set 3: OR + LA1 + SH + LA2 feature set with local averages.

Augmentation is done after generation of the spectral features dataset based on the proposed feature extraction approach. The augmentation workflow is illustrated in Figure 5 and in Table 2, Table 3, Table 4, Table 5, Table 6 and Table 7. As seen in the Figure 6, there exist two main techniques applied in the study to generate augmentation data. These are down-sampling by local averaging and shuffling. To walk-through the augmentation process, Figure 6, Figure 7, Figure 8, Figure 9, Figure 10 and Figure 11 representations based on Figure 5 are described next.

The extracted spectral features dataset is represented by Figure 6. Figure 7 shows the column-wise local average generating process. Figure 8 contains both data in Figure 6 and locally averaged data which appears at the tail end. Figure 9 shows the data in Figure 8 that has been shuffled row-wise to create variation in local averaging in the next local averaging process seen in Figure 10. Figure 11 shows an augmented dataset resulting from the original spectral features set, local averaged data, shuffling process and another local averaging process. The next paragraph puts into context the data representation in Figure 6, Figure 7, Figure 8, Figure 9, Figure 10 and Figure 11 with respect to the augmentation procedure.

If we consider an initial feature set as a matrix of data points, with each row representing a sample containing the least acceleration PSD data (SA*i*), largest acceleration PSD data (LeA*i*), least gyroscope PSD data (SG*i*), largest gyroscope PSD data (LG*i*) where *i* is the sample number, then data can be down sampled by averaging the columns to formulate the augmentation data (MSA1, MLeA1, MSG1, MLG1) … (MSA*l*, MLeA*l*, MSG*l*, MLG*l*) seen in Figure 8. In this study, we first down sample the four items continuously for all columns in Figure 7 for formulating the new samples to append to the feature set. The new feature set seen in Figure 8 is then shuffled, and down sampled again by averaging only two column items continuously for all the columns as seen in Figure 10. The augmentation data with feature vectors (Mc_11_, Mc_12_, Md_11_, Md_12_) … (Mc_K1_, Mc_K2_, Md_K1_, Md_K2_) are then appended to the shuffled data represented in Figure 8 to formulate the augmented data in Figure 11.

It should be noted that the augmentation process followed in this article is done for each class independently. The following notations and descriptions are used in this analysis: OR—Original spectral features, LA1—1st local averaging, SH—shuffling, LA2—2nd local averaging, ST—Standing, SI—Sitting, and WA—Walking.

Shuffling is done in machine learning systems to reduce on the variance of data especially when dealing with mini batch processing hence making the model to remain general and overfit less [50]. For the study experiment, row-wise random shuffling was used. The random shuffles were performed for each class exclusively.

**Figure 12:**
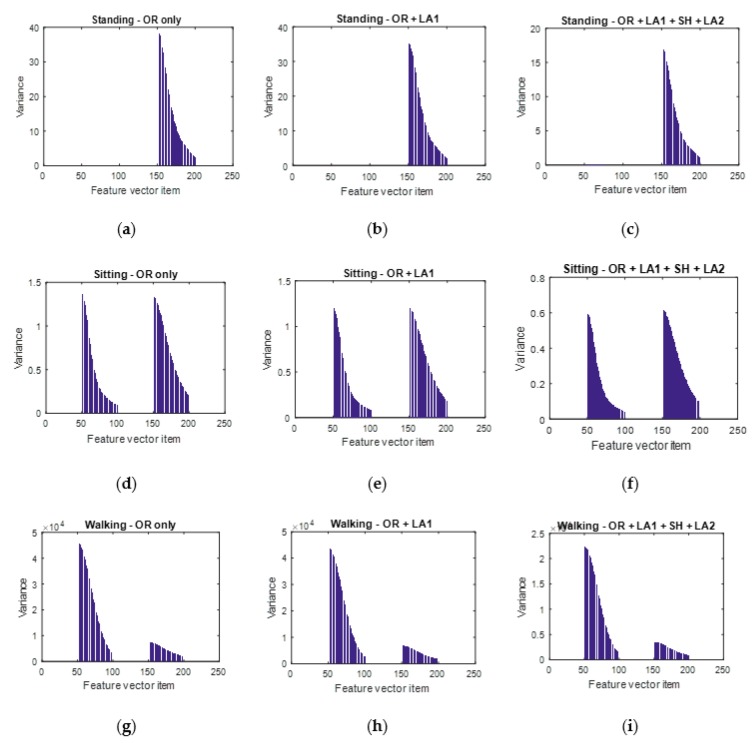
Visualizing the variance of data to check the influence of each data augmentation block. (**a**,**b**,**c**) represent the variance of the unaugmented dataset, augmented dataset after the first local averaging and that of the augmented dataset after the first local averaging, shuffling and the second local averaging procedure respectively for the standing activity. (**d**,**e**,(**f**) represent the variance of the unaugmented dataset, augmented dataset after the first local averaging and that of the augmented dataset after the first local averaging, shuffling and the second local averaging procedure respectively for the sitting activity. (**g**,**h**,**i**) represent the variance of the unaugmented dataset, augmented dataset after the first local averaging and that of the augmented dataset after the first local averaging, shuffling and the second local averaging procedure respectively for the walking activity.

The results in the variance shifts after each augmentation approach are shown in Figure 12. Figure 12b,c,e,f,h,i all shows a reduced variance in the augmented data. This is phenomenon is desirable to reduce on overfitting.

In order to further analyse the observations in Figure 12, a description of the feature vector structure is done in this paragraph. We shall use S and L as 50 for illustration. If $Q_{j},\ldots ,Q_{n}$ are the items of a feature vector $u_{i}$, then the feature vector items $Q_{1},\ldots ,Q_{n}$ are structured as follows: (1) $Q_{1},\ldots ,Q_{50}$ are the least 50 spectral densities from the accelerations spectrogram, (2) $Q_{51},\ldots ,Q_{100}$ are the largest 50 spectral densities from the accelerations spectrogram, (3) $Q_{101},\ldots ,Q_{150}$ are the least 50 spectral densities from the gyroscopes spectrogram, (4) $Q_{151},\ldots ,Q_{200}$ are the least 50 spectral densities from the gyroscopes spectrogram.

In Figure 12a–c, it is observed that only the largest part of the gyroscope’s spectral information is represented. The implication of this observation is that the huge signal wave forms in a standing scenario is mainly generated by the gyroscope but not the accelerometer. Worth noting is that our standing activity scenario was indeed one without much motion by the arm apart from some few turns.

## 4. LSTM Overview and Experimental Setup

### 4.1. LSTM Overview

**Figure 13:**
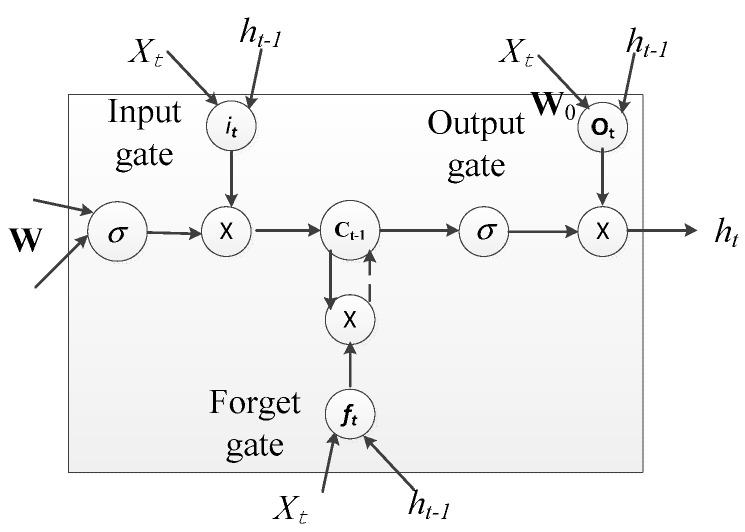
LSTM cell.

In this section, we describe the deep learning model (recurrent neural network (RNN)-LSTM model) that is used for activity recognition in this study. LSTM is a composition from RNN and can as well learn complex temporal dynamics by mapping input sequences to a sequence of hidden states and hidden states to outputs. The niche in LSTM is its ability to learn long term dependencies. In RNN the output responses $h_{i}$ are calculated based on the inputs $x_{i}$ and the responses $h_{i-1}$ from the previous time slot:

(3) $$h_{t}=\theta \left(W_{xh}X_{t}+W_{hh}h_{t-1}+b_{h}\right)$$

where θ(·) denotes the activation function, $b_{h}$ is the bias vector, $W_{xh}$ is the matrix of the weights between the input and hidden layer and $W_{hh}$ is the matrix of recurrent weights from the hidden layer to itself at adjacent time steps which is used for exploring temporal dependency. An LSTM cell is equipped with an input gate $i_{t}$, forget gate $f_{t}$, a cell $c_{t}$ and output response $h_{t}$ all defined as follows based on Figure 13:

(4) $$\begin{array}{l} i_{t}=\sigma (W_{xi}x_{t}+W_{hi}c_{t-1}+b_{i})\\ f_{t}=\sigma (W_{xf}x_{t}+W_{hf}h_{t-1}+W_{cf}c_{t-1}+bf)\\ c_{t}=f_{t}⊗c_{t-1}+i_{t}⊗\tanh(W_{xc}x_{t}+W_{hc}h_{t-1}+b_{c})\\ o_{t}=\sigma (W_{xo}x_{t}+W_{ho}h_{t-1}+W_{co}c_{t}+b_{o}),\\ h_{t}=o_{t}⊗\tanh(c_{t}), \end{array}$$

where ⊗ denotes the elementwise product, σ(x) is the sigmoid function defined as $\sigma (x)=1/(1+e^{-x})$, $W_{\alpha \beta }$ is the weight matrix between α and β (e.g., $W_{xi}$ is the weight matrix from the inputs $x_{t}$ to the gates $i_{t}$), and $b_{\beta }$ denotes the bias term of β with β∈{i, f, c, o}. The forget cell serves a major purpose of ensuring and keeping track of long term dependencies without being affected by the vanishing gradient problem exhibited during training in the traditional RNN. 

The training process for an LSTM network is based on the back-propagation process with the objective of minimizing the error. In the training process, propagation is done towards the last hidden nodes and backwards until the set number of iterations has reached. After this stage, propagation shifts to the SoftMax layer for classification. Each LSTM is stacked with 5 LSTM layers. The neural network performance is evaluated using the performance metrics shown in Table 2. The training parameters for the neural network and their values are stated in Table 3. 

The LSTM neural network is comprised of 15 hidden layers. This is considered a small number to take care of the neural network training time. The learning rates are fixed to values between 0 and 1 but closer to 0 because lower learning rates have higher chances of producing higher accuracies. Again, the learning rates are is set between 0 and 1 because the significant values of the RELU activation function exist between 0 and 1. The mini batch size is fixed because batch processing is used to improve on the training speed. The L2 regularization function is used to control the overfitting of data. The Adam optimizer is used for training the neural network through back propagation. In order to build and test the learning model, training samples and testing samples were collected. A sample in each case is made up of either a 100-feature vector or a 200-feature vector.

### 4.2. Data Collection Setup

The accelerometer and gyroscope 3D data were collected from five subjects of ages between 25 and 40 with the IMU sensor tied on their left wrist like a wrist watch. The data transmission rate for the IMU data transmission was set to 10 Hz. The raw data collected is then manipulated through the feature extraction process described in Section 3.1 to form either a 100-feature vector dataset or a 200-feature vector dataset. These 100-feature and 200-feature vector dataset are our dataset in this article.

## 5. Results and Discussion

To understand the performance of our proposed algorithms, we show in this subsection the influence of the different parts of the algorithms. First, we fix the batch size for our experiments by arbitrarily using four batch sizes (see Table 4) which are all tested on our 200-feature vector dataset and then pick out the batch size that provides the best accuracy while using 0.0002 as the learning rate.

### 5.1. Initial Feature Set Performance

We check the performance of the feature extraction algorithm without any data augmentation by varying the size of the feature vector. We collected our own 3D raw accelerometer and gyroscope data for walking, sitting and standing. Our proposed feature extraction approach described in Section 3.1 is applied to the raw data and generates a 100 and a 200-feature vector dataset which we use for analysis. The two feature sizes have been strategically chosen to represent a small or limited dataset and another with a fairly well represented data set. This serves well in testing how good a feature set is and fit for comparative testing of augmentation performance.

**Figure 14:**
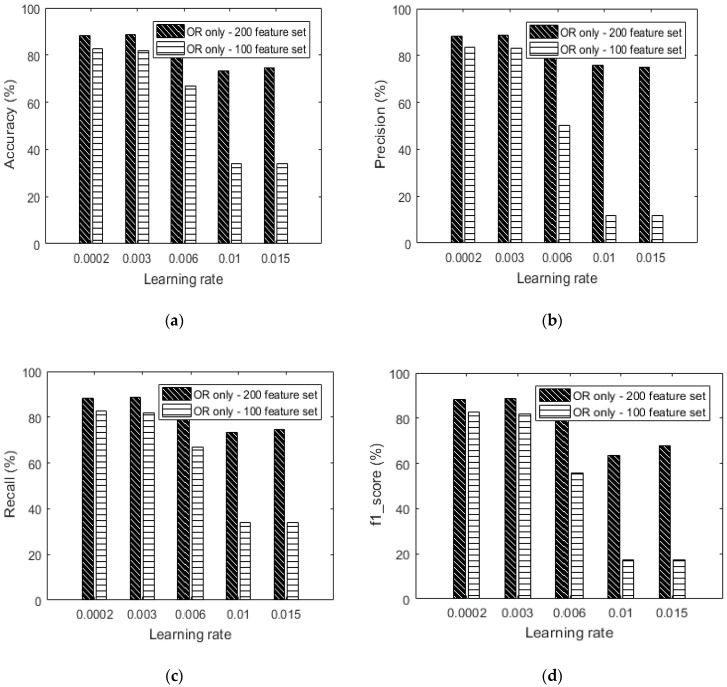
(**a**) Accuracy versus learning rate based on only the OR dataset without augmentation, (**b**) precision versus learning rate based on only the OR dataset without augmentation, (**c**) recall versus learning rate based on only the OR dataset without augmentation and (**d**) f1_score versus learning rate based on only the OR dataset without augmentation.

Figure 14 shows that the 200-feature vector dataset performs better than the 100-feature vector at all learning rates with the best accuracy at 88.7% and a learning rate of 0.003. This performance phenomenon is in tandem with the notion that learning with more features should offer a higher degree of accuracy.

Table 5 shows the results from using only OR (without augmentation) with varying learning rates for the 100-feature vector dataset. It should be noted here that the performance of the 100-feature data set is very bad when learning rates of 0.01 and 0.015 are applied. By observing the confusion matrices, it can be said that the dataset is either unbalanced or the size of the dataset is too small for learning to be achieved. It is for such reasons that we intend to show the effect of performing data augmentation on such a dataset. Figure 14 shows the detailed performance of the 100-feature dataset with various performance indicators.

Table 6 shows the results from using only OR (without augmentation) with varying learning rates for the 200-feature vector dataset. By observing the confusion matrices, the 200-feature vector dataset exhibits a more balanced dataset compared to the 100-feature vector dataset. At least all classes are recognizable using all the learning rates. It can be concluded that the greater vector size in a feature set contributed to the accuracy performance seen in Figure 14.

The 100 spectral features dataset exhibits a lesser training time of about 1 h and 45 min compared to about 2 h and 30 min for the larger 200 feature dataset.

### 5.2. Data Augmentation Performance

Each feature vector size is analyzed independently to understand the performance of each augmentation block. We start with the 100-feature vector dataset. Table 7 shows the recognition results of the dataset augmented by the local average (where G = 4). Compared to the confusion matrices earlier represented in Table 5 of the same dataset, it is evident that Table 7 presents more balanced dataset especially for Table 7b,d,e with reference to Table 5b,d,e.

**Figure 15:**
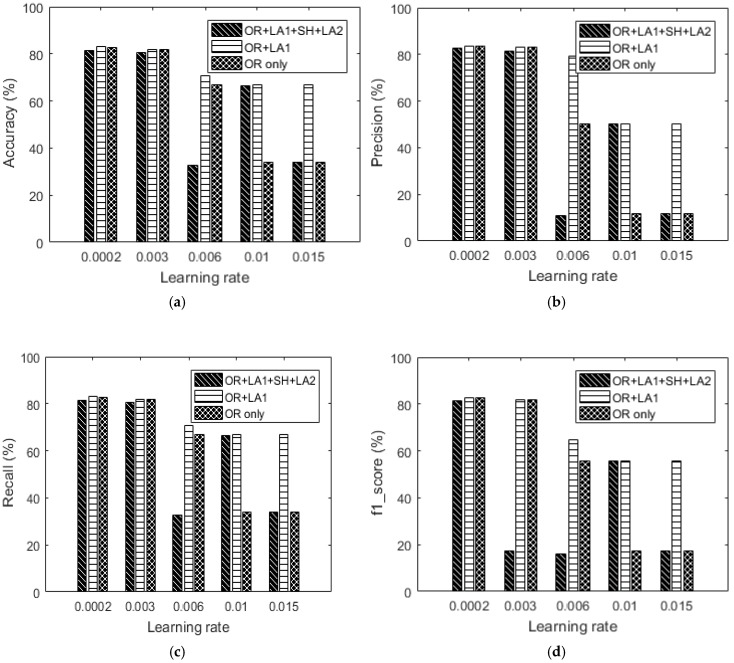
100-feature vector dataset: (**a**) Accuracy versus learning rate, (**b**) precision versus learning rate, (**c**) recall versus learning rate and (**d**) f1_score versus learning rate.

Next, we show the influence of data augmentation by OR + LA1 + SH + LA2. Table 8 shows the confusion matrices and Figure 15 includes the summarized results for OR + LA1 + SH + LA2. It should be noted that the LA2 after shuffling was done with G specified as G = 2. The results of this augmentation show that lower learning rates results into approximately similar recognition accuracies, however for higher learning rates, of 0.006, 0.01 and 0.015 the accuracy is significantly low compared to both the OR and OR + LA1 results. From this observation, it can be concluded that the parameter G in our augmentation algorithm is critical in determining augmentation performance by local averaging.

**Figure 16:**
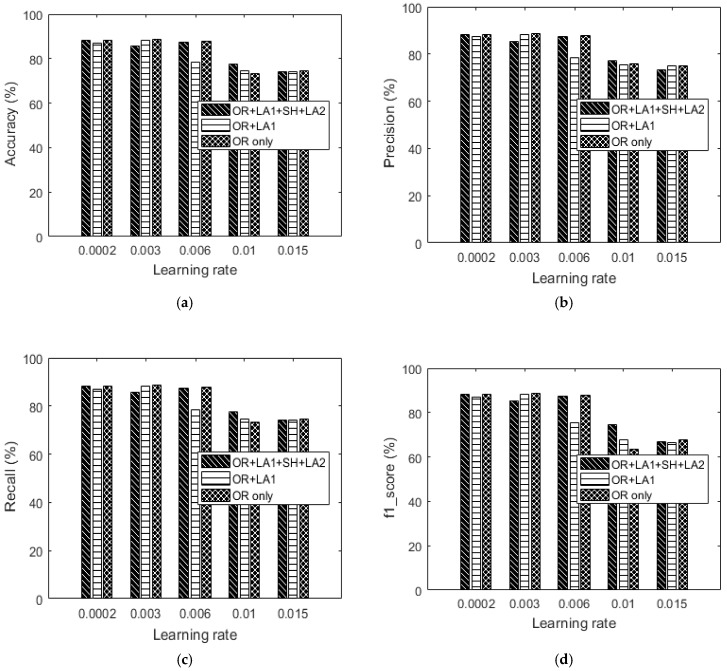
200-feature vector dataset: (**a**) Accuracy versus learning rate, (**b**) precision versus learning rate, (**c**) recall versus learning rate and (**d**) f1_score versus learning rate.

Summarily, by comparing the accuracy results in Figure 16 to the OR only results in the same Figure 16, the OR + LA1 augmentation achieves significant accuracy improvement by 32.75% for 0.01 learning rate, 32.7% for 0.015 learning rate, and 3.89% for 0.006 learning rate.

The augmentation performance of the 200-feature vector dataset is now considered. Preliminary results (Table 6 and Figure 14) without augmentation show that the data imbalance problem isn’t significant in the dataset. Table 9 and Table 10 show the confusion matrices for the dataset augmented by only local averaging (where G = 4 in LA1 and G = 2 in LA2). The results in Figure 16 show that our augmentations provide almost similar results for low learning rates at 0.0002 and 0.003. However, at higher learning rates OR + LA1 competes both OR and OR + LA1 + SH + LA2. Table 10 shows the confusion matrices for the OR + LA1 + SH + LA2—200-feature vector dataset.

By comparing OR and OR + LA1 + SH + LA2 results in Figure 16 respectively, it should be noted that at a learning rate of 0.01, a significant improvement of up to 4.24% in accuracy is observed. This observation is also true for the 100-feature vector dataset exhibiting 32.75% improvement in accuracy.

Summarily, the effect of our augmentation approach is seen to be more effective in the lesser feature vector size dataset. The approach is seen to help improve on lower recognition accuracy caused by the data imbalance problem.

### 5.3. Validation of Proposed Algorithm

In this section, we implement the proposed data augmentation approaches exclusively on a HAR dataset available from the University of California (UCI) machine learning repository [10] to check on its performance. The specific data used in this validation is an extract of the three activities (standing, sitting and walking) from the original dataset. Our intention is to try as much as possible to compare the quality of our dataset with a state-of-the-art dataset based on the proposed feature extractions and augmentations. Again, selecting only three specific activities from the original dataset is fit for pinpointing the unbalanced data problem which is common in discriminating classes ranging from 2 to 3. It is therefore expected that an unbalanced class scenario shall manifest in the experiments hence the need for solutions such as data augmentations. Therefore 3886 training series and 1519 test series were extracted and trained using the same LSTM network settings that are used for training our own datasets. The UCI dataset is a 128-feature vector dataset. The training and test data for the accelerometer and gyroscope were used.

**Figure 17:**
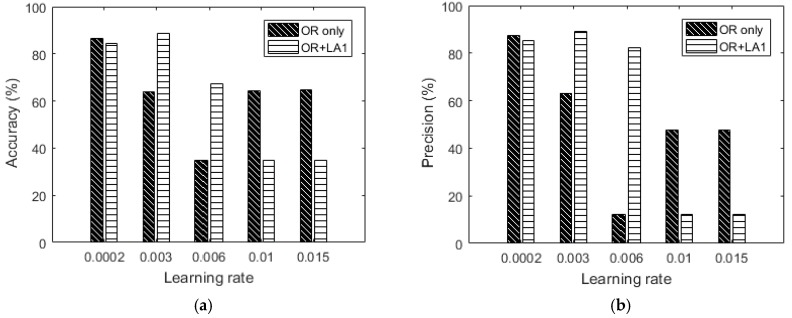

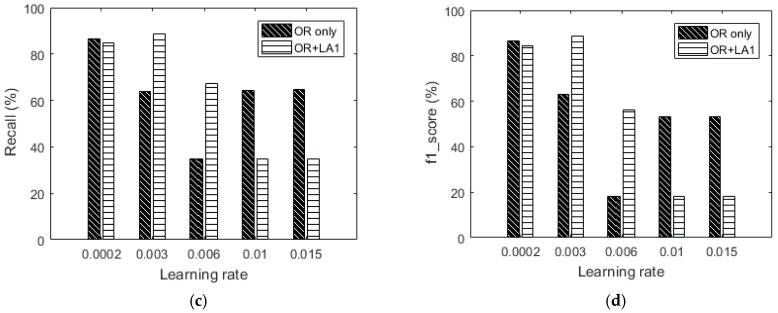
UCI’s 128-feature vector dataset: (**a**) Accuracy versus learning rate, (**b**) precision versus learning rate, (**c**) recall versus learning rate and (**d**) f1_score versus learning rate.

**Figure 18:**
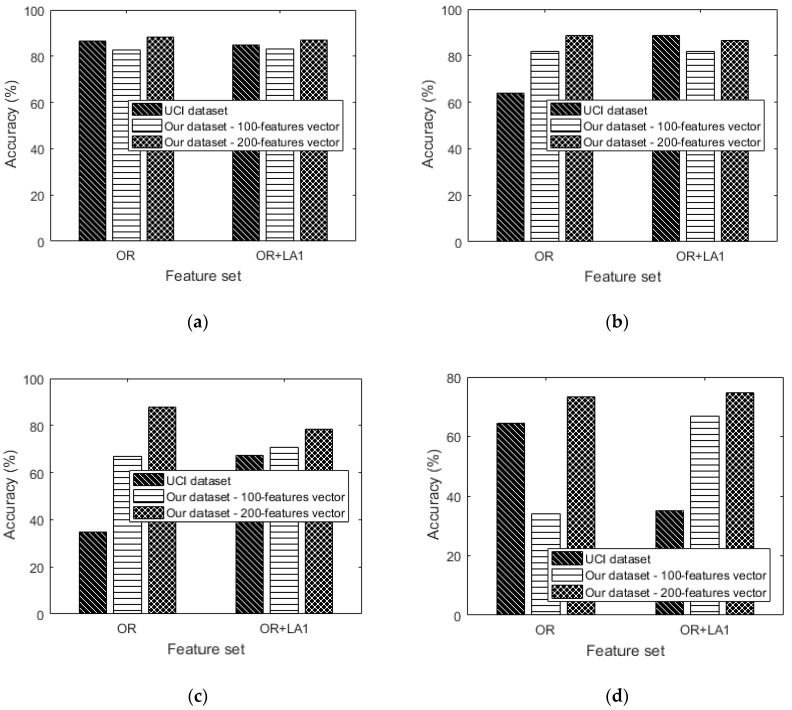
Comparing the performance of OR and OR + LA1 using the UCI dataset and our dataset at various learning rates of: (**a**) 0.0002 (**b**) 0.003 (**c**) 0.006 and (**d**) 0.01.

Preliminary augmentation results from our own dataset, showed that OR + LA1 is better than OR + LA1 + SH + LA2. We have therefore chosen to test OR and OR + LA1 for our validation. Note that: (1) OR for the UCI dataset represents the original online dataset without any manipulation by the authors of this paper. (2) The OR + LA1 for the UCI dataset represents the original UCI online dataset which the authors in this paper have manipulated by performing the first local averaging procedure refered to as LA1 in this paper and then used as augmentation data. Table 11 and Table 12 show the OR+ LA1 and OR only confusion matrix results. The graphical results on accuracy, precision, recall and f1_score are shown in Figure 17 and Figure 18. Both OR and OR + LA1 performance results are shown in Figure 17 and Figure 18 for comparison. 

Based on Figure 17, the best accuracy result from the UCI HAR dataset (OR—without augmentation) is 86.7%. With augmentation, the best recognition accuracy is 88.87%. Although this improvement of 1.27% on accuracy is small, the proposed approach gives a significant improvement of 24.92% on accuracy using a learning rate of 0.003. The same learning rate of 0.003 generates the best accuracy and is based on the OR + LA1 augmentation. It can be observed in Table 12b,d,e that the classification is skewed towards some class or classes. The same phenomenon can be seen in Table 11b,d,e. 

Figure 18 shows that OR + LA1 augmentation is competitive on accuracy at low learning rates when compared to using only OR for classification. Figure 18 shows the results of accuracy for both the UCI dataset and our dataset. In Figure 18a, our 200-feature vector dataset outperforms the UCI dataset in both OR and OR + LA1. This could be a result of the greater size of the feature vector. In Figure 18b, Our dataset without any augmentation outperforms the original UCI dataset for both the 100 and 200-feature vectors dataset by 18% and 24.81% in accuracy respectively. OR + LA1 in Figure 18b for the UCI dataset shows an improvement in accuracy compared to its OR accuracy result. This shows that OR + LA1 augmentation has had a positive effect on improving the accuracy by 24.95% for the UCI dataset.

In Figure 18c, our dataset without any augmentation outperforms the UCI dataset for both the 100 and 200-feature vectors by 31.74% and 52.77% respectively. In the OR + LA1 scenario, our dataset out performs the UCI dataset for both the 100 and 200-feature vector dataset by 3.37% and 11.1% respectively. We can also observe in Figure 18c that the OR + LA1 augmentation caused an improvement in the UCI dataset by 32.6% in accuracy.

In Figure 18d, the performance based on OR is poor for our 100-feature vector dataset compared to both the UCI dataset and the 200-feature vector dataset. This is likely to be an effect of the larger learning rate applied in a small size dataset. In OR + LA1 of Figure 18d, the 200-feature vector dataset relatively maintained its accuracy level while the UCI dataset declined in accuracy. It is also observed that the 100-feature vector dataset improved in accuracy in the OR + LA1 scenario. Generally, Figure 18d especially for the UCI dataset provides insights that, higher learning rates may require careful feature representation especially when data augmentation is a must.

## 6. Conclusions and Future Work

In this paper, we proposed using only spectral features for learning human activity. We showed that using few spectral features we can achieve state-of-the-art recognition performance. Fewer spectral features exhibit a lesser training time of about 1 h and 45 min compared to about 2 h and 30 min for the large feature set used in this article. Furthermore, an augmentation ensemble used in feature space was also defined for purposes of improving recognition accuracy. Through greedy tuning of the learning rate, our proposed feature extraction and augmentation ensemble achieved improved recognition accuracy at several learning rates and in a few cases, performs close to the unaugmented feature set. The proposed extraction approach provided the best performance improvement in accuracy of 52.77% in comparison with the UCI online dataset. The proposed OR + LA1 provided the best performance improvement in accuracy of 32.6% compared with the UCI online dataset. The LSTM deep learning model was utilized in the study. Therefore, further studies in this work shall be done to compare the performance of the proposed approach on other HAR datasets and various machine learning models.

## Figures and Tables

**Table 1:** Some of the most widely used features as discussed in [34].

Type	Features	Some Applications
Time-domain	Mean [35], variance, standard deviation [36,37], root mean square [38,39], zero or mean crossing rate [40], derivative, peak counts [41,42]	Human activity recognition [37,38], speech recognition [39], eye movement analysis [42]
Frequency-domain	Discrete fast Fourier transforms coefficient, spectral energy [7,43]	Human activity recognition [7]
Time frequency domain	Wavelet coefficients [44]	Blink detection [42]

**Table 2:** Performance metrics.

Acronym	Description
Accuracy	The percentage of correctly predicted samples out of the total number of samples.
Precision	The fraction of the samples which are actually positive among all the samples which we predicted positive. $Precision=\frac{N_{TP}}{N_{PP}}$ where, $N_{TP}$ is the number of true positives and $N_{PP}$ is the number of predicted positives.
Recall	Measures the proportion of positives that are correctly identified. $Recall=\frac{N_{TP}}{N_{AP}}$ Where, $N_{TP}$ is the number of true positives and $N_{AP}$ is the actual number of positives.
f1_score	The weighted harmonic means of precision and recall.

**Table 3:** Experimental setup.

Description	Value
Number of hidden nodes	15
Learning rates tested	0.002, 0.006, 0.003, 0.015, 0.01
Mini batch size	8000
Loss	0.001
Regularization	L2
Activation function	RELU (rectified linear unit)
Number of training samples (OR + LA1 + SH + LA2)	9616
Number of training samples (OR + LA1)	6414
Number of training samples (OR only)	5132
Number of test samples	2614
Optimization (back propagation)	Adam optimizer

**Table 4:** Batch size versus accuracy (OR + LA1 + SH + LA2).

Batch Size	Accuracy
4500	76.95
4000	80.51
6000	82.8
8000	88.14

**Table 5:** Confusion matrices: (OR only)—100-feature vector datasets.

Learning Rate		(a) 0.0002			(b) 0.006			(c) 0.003			(d) 0.015			(e) 0.01		
True label	ST	441	240	0	0	681	0	409	272	0	0	0	681	0	0	681
	SI	113	560	0	0	673	0	98	575	0	0	0	673	0	0	673
	WA	0	1	700	0	2	699	0	1	700	0	0	701	0	0	701
		ST	SI	WA	ST	SI	WA	ST	SI	WA	ST	SI	WA	ST	SI	WA
		Predicted label														

**Table 6:** Confusion matrices: (OR only)—200-feature vector dataset.

Learning Rate		(a) 0.0002			(b) 0.006			(c) 0.003			(d) 0.015			(e) 0.01		
True label	ST	547	153	0	550	149	1	555	145	0	81	580	39	13	656	31
	SI	154	1066	0	168	1052	0	150	1070	0	33	1177	10	3	1207	10
	WA	0	0	694	1	0	693	0	0	694	0	0	694	1	0	693
		ST	SI	WA	ST	SI	WA	ST	SI	WA	ST	SI	WA	ST	SI	WA
		Predicted label														

**Table 7:** Confusion matrices: (OR + LA1)—100-feature vector dataset.

Learning Rate		(a) 0.0002			(b) 0.006			(c) 0.003			(d) 0.015			(e) 0.01		
True label	ST	437	244	0	97	584	0	416	265	0	0	681	0	0	680	1
	SI	106	567	0	18	655	0	103	570	0	0	673	0	0	673	0
	WA	0	1	700	1	0	700	0	1	700	0	1	700	0	0	701
		ST	SI	WA	ST	SI	WA	ST	SI	WA	ST	SI	WA	ST	SI	WA
		Predicted label														

**Table 8:** Confusion matrices: (OR + LA1 + SH + LA2)—100-feature vector dataset.

Learning Rate		(a) 0.0002			(b) 0.006			(c) 0.003			(d) 0.015			(e) 0.01		
True label	ST	406	273	2	0	681	0	391	289	1	0	0	681	0	681	0
	SI	91	578	4	0	678	0	102	571	0	0	0	673	0	673	0
	WA	7	2	692	0	701	0	10	0	691	0	0	701	0	8	693
		ST	SI	WA	ST	SI	WA	ST	SI	WA	ST	SI	WA	ST	SI	WA
		Predicted label														

**Table 9:** Confusion matrices: (OR + LA1)—200-feature vector dataset.

Learning Rate		(a) 0.0002			(b) 0.006			(c) 0.003			(d) 0.015			(e) 0.01		
True label	ST	578	121	1	223	477	0	537	168	0	56	611	33	56	611	33
	SI	219	1001	0	88	1132	0	140	1080	0	21	1188	11	21	1188	11
	WA	0	0	694	0	0	694	0	0	694	1	0	693	1	0	693
		ST	SI	WA	ST	SI	WA	ST	SI	WA	ST	SI	WA	ST	SI	WA
		Predicted label														

**Table 10:** Confusion matrices: (OR + LA1 + SH + LA2)—200-feature vector dataset.

Learning Rate		(a) 0.0002			(b) 0.006			(c) 0.003			(d) 0.015			(e) 0.01		
True label	ST	406	273	2	0	681	0	391	289	1	0	0	681	0	681	0
	SI	91	578	4	0	678	0	102	571	0	0	0	673	0	673	0
	WA	7	2	692	0	701	0	10	0	691	0	0	701	0	8	693
		ST	SI	WA	ST	SI	WA	ST	SI	WA	ST	SI	WA	ST	SI	WA
		Predicted label														

**Table 11:** Confusion matrices: (OR + LA1).

Learning Rate		(a) 0.0002			(b) 0.006			(c) 0.003			(d) 0.015			(e) 0.01		
True label	ST	455	67	10	525	0	7	471	57	4	532	0	0	532	0	0
	SI	153	337	1	485	1	5	107	383	1	491	0	0	491	0	0
	WA	0	0	496	0	0	496	0	0	496	496	0	0	496	0	0
		ST	SI	WA	ST	SI	WA	ST	SI	WA	ST	SI	WA	ST	SI	WA
		Predicted label														

**Table 12:** Confusion matrices: (OR only).

Learning Rate		(a) 0.0002			(b) 0.006			(c) 0.003			(d) 0.015			(e) 0.01		
True label	ST	480	48	4	532	0	0	319	210	3	0	528	4	0	530	2
	SI	145	344	2	491	0	0	325	160	6	0	486	5	0	486	5
	WA	3	0	493	496	0	0	0	4	492	0	0	496	0	2	494
		ST	SI	WA	ST	SI	WA	ST	SI	WA	ST	SI	WA	ST	SI	WA
		Predicted label														
